# Diagnostic performance of a novel ESAT6-CFP10 skin test for tuberculosis infection in school tuberculosis outbreak in China

**DOI:** 10.3389/fpubh.2023.1259106

**Published:** 2024-01-12

**Authors:** Peng Lu, Jingjing Xu, Rong Wang, Xiaona Gong, Qiao Liu, Xiaoyan Ding, Wei Lu, Limei Zhu

**Affiliations:** ^1^Department of Chronic Communicable Disease, Center for Disease Control and Prevention of Jiangsu Province, Nanjing, Jiangsu, China; ^2^Center for Disease Control and Prevention of Yancheng City, Yancheng, Jiangsu, China; ^3^School of Public Health, Nanjing Medical University, Nanjing, Jiangsu, China; ^4^Center for Disease Control and Prevention of Nanjing City, Nanjing, Jiangsu, China; ^5^Center for Disease Control and Prevention of Lishui District, Nanjing, Jiangsu, China

**Keywords:** ESAT6-CFP10 skin test, tuberculosis, school, outbreak, computerized tomography

## Abstract

**Background:**

The ESAT6-CFP10 (EC) skin test is recommended by the World Health Organization for latent tuberculosis infection (LTBI). However, it is still unknown how the EC skin test performs in students during a school tuberculosis outbreak.

**Methods:**

We conducted an epidemiological investigation to assess the performance of the EC skin test in this high-risk population.

**Results:**

A total of 9 active student patients were confirmed in the same class as the index case, with an incidence rate of 18.0% (9/50). Among the 50 close contacts, 14 (28%) were over 15 years old and had a chest X-ray (CXR), and none of them had abnormal CXR findings. The rates of positive tuberculin skin test (TST) ≥ 5 mm and < 10 mm, ≥ 10 mm and < 15 mm, and ≥ 15 mm were 12.0% (6/50), 16.0% (8/50), and 10.0% (5/50), respectively. On the second screening, 44 students with the same class as the index case had the EC skin test, of which 31 (70.5%) had positive EC tests. All patients had negative sputum smear results, of whom 4 (44.4%) had positive Xpert results; three had a TST induration diameter between 5 mm and 10 mm, but all of them had an EC diameter > 15 mm; 5 (55.6%) had abnormal CXR results, but all the confirmed patients had abnormal CT results; Except for four cases that were diagnosed by Xpert, the remaining five were confirmed by CT scan.

**Conclusion:**

The novel EC skin test performed well in students during the school tuberculosis outbreak. In some special conditions, such as when the index case is bacteriologically positive for tuberculosis and the rate of LTBI is higher than the average for the local same-age group, secondary screening is recommended 2–3 months after the first screening. Furthermore, we cannot ignore the role of CT in the diagnosis of early student tuberculosis.

## Background

School tuberculosis outbreaks are common and always raise public health concerns in China. One of the most important reasons is that students who are recently infected with latent tuberculosis infection (LTBI) often refuse to have preventive treatments (1). The tuberculin skin test (TST) is the most widely used test for LTBI. In most countries, a TST induration of 5 mm or more is considered positive and indicates that the person has been infected with *Mycobacterium* tuberculosis. In China, a TST induration of 15 mm or more is considered positive and indicates that the person is at high risk of developing active tuberculosis (2). Because, it is difficult to explain the results of the TST when only students with an induration diameter of ≥15 mm, such as blister, necrosis, and lymphangitis, need to receive preventive treatment. Because, for students with an induration diameter of 5–15 mm, nothing is done but to review their chest X-ray (CXR) after 3 months (3).

Therefore, a simple and easily understandable test is urgently needed in school tuberculosis outbreaks. In 2022, the World Health Organization (WHO) recommended newer *Mycobacterium* tuberculosis antigen-based skin tests as alternatives to diagnose LTBI, including Cy-Tb (Serum Institute of India, India), Diaskintest (Generium, Russian Federation), and C-TST (formerly known as ESAT6-CFP10 (EC) test, Anhui Zhifei Longcom, China) (4). In recent studies, the EC skin test has shown superiority in diagnostic value to the TST, which was similar to the interferon-gamma release assay (IGRA) among the general population (5). The positive cutoff value of the EC skin test is 5 mm, which suggests that EC may play an important role in addressing school tuberculosis epidemics. However, it is still unknown how the EC skin test performs in students during a school tuberculosis outbreak.

To address this question, we recruited students in a school tuberculosis outbreak in Jiangsu Province to assess the performance of the EC skin test in this high-risk population.

## Methods

On January 1, 2022, a 15-year-old student was diagnosed with active tuberculosis after a sputum smear microscopy result of 2+. The patient self-reported that they had been coughing for more than 20 days and had also experienced night sweats. Epidemiological and contact investigations were conducted in the patient's class to determine the source of the infection.

All the processes of the epidemiological investigation were conducted in accordance with the guidelines of the Chinese School Tuberculosis Control and Prevention Technical Work Regulations (6). After an active case of tuberculosis was found in a student, the staff of the Center for Disease Control and Prevention (CDC) immediately conducted epidemiological investigations.

First, they conducted TSTs and CXRs among close contacts of the index case. If one or more new cases of tuberculosis were found during this initial screening, or the strong positive rate of TST detection in close contacts was significantly higher than that in the same age group in the area, the scope of contact screening would be expanded to include all general contacts.

If one or more new cases of tuberculosis were found in the expanded screening, or the strong positive rate of TST detection in close contacts was still significantly higher than that in the same age group in the area, the scope of contact screening would be expanded to include the entire school population.

This process of expanding the scope of contact screening is based on the principle of contact tracing, which is the process of identifying and following up with people who have been in close contact with a person who has a contagious disease. Contact tracing is an important tool for preventing the spread of tuberculosis and other infectious diseases.

Considering that most of the students were under 15 years old, symptom consultation and TSTs were conducted first. Students with fever, weight loss, fatigue, cough, sputum, etc., and an induration diameter of ≥15 mm would have a CXR. Individuals with abnormal CXR scans were transferred to a local designated tuberculosis hospital for further diagnosis, including smear microscopy and GeneXpert MTB/RIF computed tomography (CT).

A positive TST was defined as an induration diameter ≥10 mm. An induration reaction of 10 ≤ TST <15 mm was defined as moderately positive, and ≥15 mm was defined as a strong positive.

According to the guidelines of the Chinese School Tuberculosis Control and Prevention Technical Work Regulations (6), students with negative TST results were recommended to repeat the TST after 3 months if there were three or more active tuberculosis patients in their class with an epidemiological link. In this school tuberculosis outbreak, EC skin tests (1.0 μg/0.1 mL) were conducted in those with an induration of TST <10 mm. A positive EC skin test was defined as an induration diameter ≥ 5 mm.

Close contacts: (1) Students and teachers who study in the same classroom or live in the same dormitory as the tuberculosis patient. (2) Family members who have had direct contact with the tuberculosis patient in the same residence for up to 7 days, from 3 months before diagnosis to 14 days after treatment begins. (3) Other patients who have had direct contact for 8 h or more, or cumulatively up to 40 h in a closed space, with a bacteria-positive, severely bacteria-negative, or obvious-symptom bacteria-negative patient from 3 months before diagnosis to 14 days after starting treatment. This also includes patients who have accumulated 40 h of exposure with other bacteria-negative patients in the 1 month before diagnosis.

General contacts: Those who study and live together on the same teaching floor or dormitory floor as the index case.

Occasional contacts: Those who study and live together in the same teaching building or dormitory building but not on the same floor as the index case, or who have occasional contact with teachers and students (6).

### Ethics approval and consent to participate

All data were collected from an epidemiological investigation conducted in accordance with the School Tuberculosis Control and Prevention Technical Work Regulations (6). Therefore, ethics approval was not required for this study. All students and their parents were informed of the school tuberculosis outbreak and the examinations that would be conducted.

### Statistical analysis

We used standard 2 × 2 contingency tables to summarize the categorical variables. All data were analyzed using SPSS software (version 23.0, IBM Corporation, Armonk, NY).

## Results

### Demographic characteristics

A total of 50 students took part in the screening. Approximately half of the participants were males (56.0%). Out of the total, 31 individuals (62%) exhibited an induration diameter of TST <5 mm, while 31 students (70.5%) tested positive in the EC skin test. All students exhibited normal chest X-ray results during the initial screening. However, upon reevaluation during the second screening, 6 students (19.4%) demonstrated a transition to abnormal chest X-ray findings, as outlined in Table 1.

**Table 1:** Demographic characteristics of the 50 students.

**Characteristics**	**All participants *N* (%)**
*N*	50 (100)
**Sex**	
Female	22 (44.0)
Male	28 (56.0)
**Tuberculin skin test (mm)**	
< 5	31 (62.0)
≥5 to < 10	6 (12.0)
≥10 to < 15	8 (16.0)
≥15	5 (10.0)
**ESAT6-CFP10 skin test (mm)**	
< 5	13 (29.5)
≥5	31 (70.5)
**Chest X-ray**	
Normal	25 (80.6)
Abnormal	6 (19.4)

### Analysis of the epidemic

A total of 9 active tuberculosis patients were confirmed in the same class as the index case, yielding an incidence rate of 18.0% (9/50). The moderate and strong positive rates of close contacts were 16.0% (8/50) and 10.0% (5/50), respectively. The positive rate of EC during the second screening was 70.5% (31/44). The tuberculosis cluster epidemic had a strong possibility of being a homologous outbreak based on the field epidemiology.

### Contact investigation

**Figure 1:**
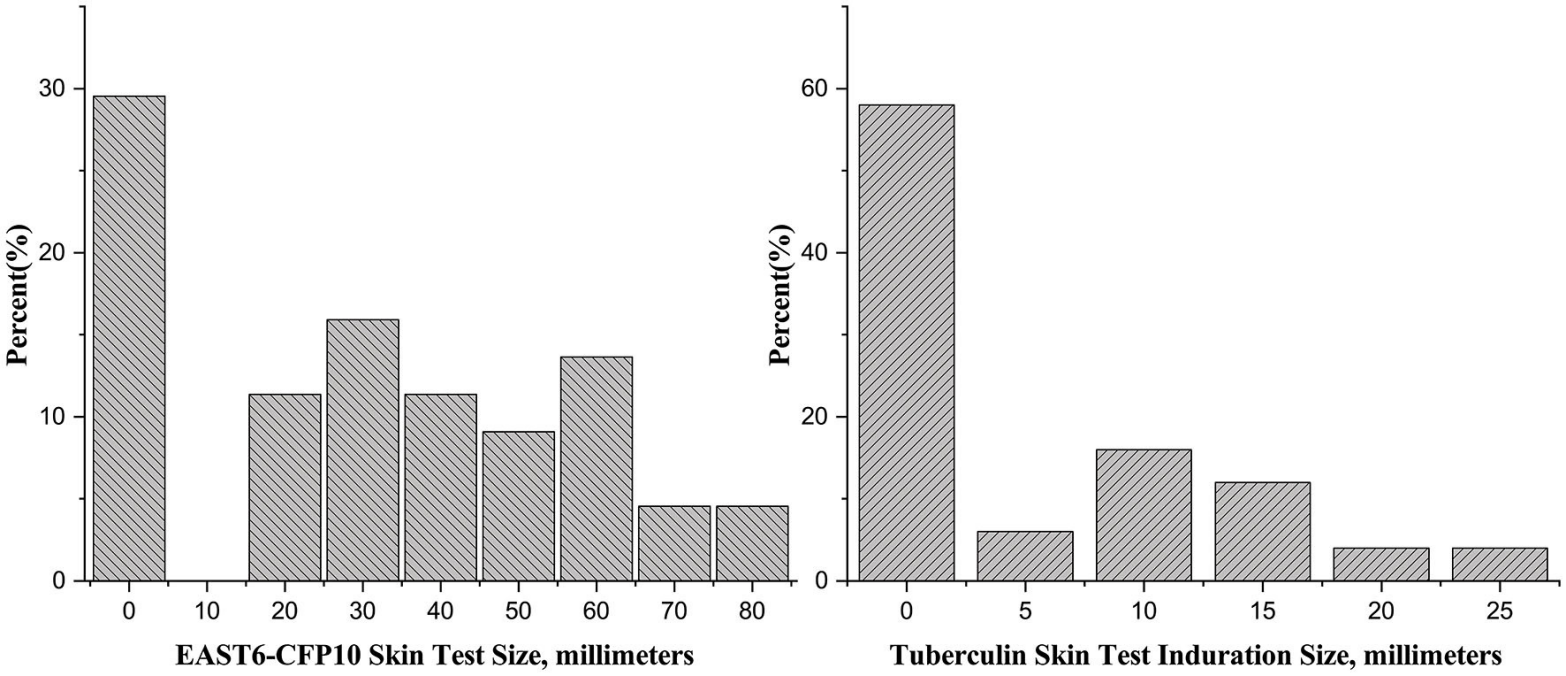
Distribution of EAST6-CFP10 skin test and tuberculin skin test.

Two teachers refused to have the TST and CXR, so a total of 40 students and 10 teachers were screened in the first round. None of them had undergone prior prophylactic testing, either through skin or immunological assessments. Among the 50 close contacts, 14 (28%) were older than 15 years old and had a CXR. None of them had abnormal CXR findings. The rates of positive TST with induration diameters of ≥5 mm and <10 mm, ≥10 mm and <15 mm, and ≥15 mm were 12.0% (6/50), 16.0% (8/50), and 10.0% (5/50), respectively (Figure 1).

In the second round, 44 students who were in the same class as the index case had the EC skin test, of which 31 (70.5%) had positive results. The mean (standard deviation, SD) was 27.4 (23.1) mm. Among the 31 individuals with positive EC tests, 7 had abnormal CXR results (Table 2).

**Table 2:** Screening of close and general contacts in the junior high school.

**Group**	**Total no**.	**TST (mm)**			**Abnormal X-ray**	**Abnormal CT scan**	**Total no**.	**EC skin test (mm)**		**Abnormal X-ray**	**Abnormal CT scan**	**Confirmed cases**
		** < 10 *N* (%)**	**10 ≤ and < 15 *N* (%)**	**≥15 *N* (%)**				** < 5 *N* (%)**	**≥5 *N* (%)**			
In the same class	50	37 (74%)	8 (16%)	5 (10%)	0	-	50	13	31	8	17	9
In the same floor	-	-	-	-	-	-	161	153	8	0	-	0

TST, tuberculin skin tests; EC, ESAT6-CFP10; No, number.

Subsequently, 173 people who shared the same floor as the index case underwent LTBI screening. Of these, 161 had the EC skin test and 12 had T-SPOT tests due to contraindications. All 12 T-SPOT tests were negative. Among the 161 individuals who had the EC skin test, 8 (5.0%) had positive results (data not shown). Among the 211 contacts, the rate of LTBI determined by the EC skin test was 18.5 (Table 2).

### Characteristics of active tuberculosis

**Figure 2:**
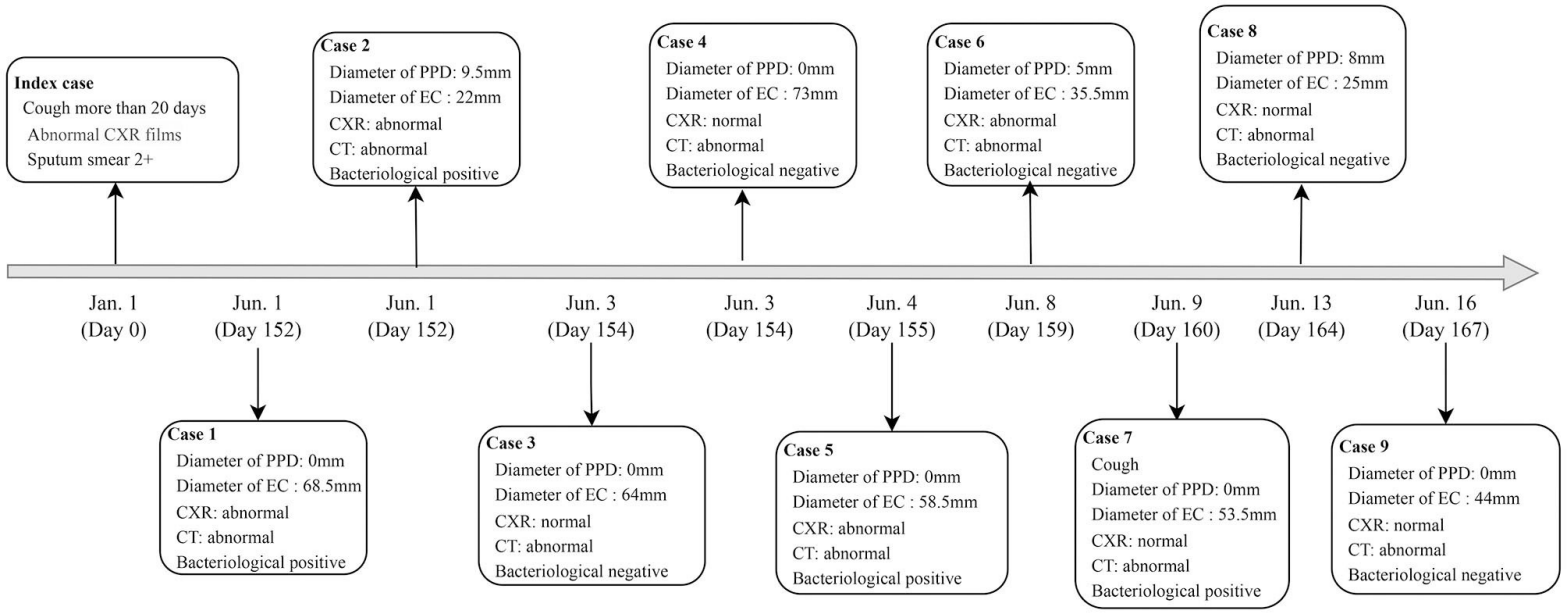
Timeline of confirmed tuberculosis cases in Jiangsu Province, China.

A total of 9 student tuberculosis cases were found in this outbreak, excluding the index case. Of these, 5 (55.6%) were male and 4 (44.4%) were female. All patients tested negative for sputum smear, with 4 individuals (44.4%) showing positive Xpert results without Rifampicin resistance. Three patients had TST induration diameters between 5 mm and 10 mm, but all of them had EC diameters >15 mm. Five patients (55.6%) had abnormal CXR results, but all confirmed patients had abnormal CT results. Four cases were diagnosed by Xpert, and the remaining 5 were confirmed by CT scan (Table 3, Figure 2).

**Table 3:** Characteristics of active tuberculosis cases identified during a school outbreak, Jiangsu, China, 2022 (*n* = 10).

**Case**	**Sex**	**Symptoms**	**Discovery approach**	**TST (mm)**	**Chest X-ray**	**EC (mm)**	**CT scan**	**Microscopy**	**Xpert**	**Date of diagnosis**
Index case	Male	Cough	Sputum Smear	-	Abnormal	-	-	2+	Positive	Jannuary 1
Case 1	Female	None	Xpert	0	Abnormal	68.5	Abnormal	Negative	Positive	June 1
Case 2	Male	None	Xpert	9.5	Abnormal	22	Abnormal	Negative	Positive	June 1
Case 3	Female	None	CT	0	Normal	64	Abnormal	Negative	Negative	June 3
Case 4	Female	None	CT	0	Normal	73	Abnormal	Negative	Negative	June 3
Case 5	Female	None	Xpert	0	Abnormal	58.5	Abnormal	Negative	Positive	June 4
Case 6	Male	None	CT	8	Abnormal	35.5	Abnormal	Negative	Negative	June 8
Case 7	Male	Cough	Xpert	0	Normal	53.5	Abnormal	Negative	Positive	June 9
Case 8	Male	None	CT	8	Normal	25	Abnormal	Negative	Negative	June 13
Case 9	Male	None	CT	0	Normal	44	Abnormal	Negative	Negative	June 16

TST, tuberculin skin tests; EC, ESAT6-CFP10; CT, computerized tomography.

22 (70.1%) left with positive EC skin test excluding active tuberculosis were all accepted tuberculosis preventive therapy (TPT), and after 1 year follow up, no active tuberculosis cases were found.

## Discussion

A school tuberculosis outbreak is a public health emergency that requires immediate attention (7–11). TST, one of the most widely used tests for LTBI, can be difficult to interpret. The WHO recommends the EC skin test for LTBI testing in 2022 because it has similar diagnostic value to IGRA tests (4, 5). To our knowledge, this is the first study to evaluate the performance of the EC skin test in a school tuberculosis outbreak. The EC skin test showed high superiority in finding student tuberculosis patients. We also found that if the index case was etiological positive for tuberculosis, close contacts should be recommended to have a CXR regardless of whether they are over 15 years old.

Unlike other countries, individuals with a TST induration diameter of ≥5 mm or ≥10 mm are regarded as LTBI and should have preventive treatment (12), China used 15 mm as the cutoff criterion of a TST to indicate preventive treatment (6). For students with a TST induration diameter between 5 mm and 15 mm, there is no other action other than enhanced follow-up if there are one or two active tuberculosis cases in the class. However, if there are three or more active tuberculosis patients in the class with an epidemiological link, then a CXR examination should be performed 3 months after the first screening (6). In contrast to the TST, the EC skin test may have innate superiority, similar to IGRA, due to the cutoff value of 5 mm (5). After excluding the possibility of tuberculosis, students with a diameter ≥5 mm of EC skin test should have preventive treatment (6). In this study, the EC skin test detected all 9 tuberculosis patients, which showed a sensitivity of 100%. Three published studies evaluated the sensitivity of the EC skin test. The pooled sensitivity at the ≥5 mm induration was 86% (95% CI: 83%−89%) (5, 13, 14). These findings suggest that the EC skin test can play an important role in the detection of tuberculosis in school outbreaks. Additionally, the EC skin test is less likely to be affected by previous bacillus Calmette Guerin (BCG) vaccination or exposure to non-tuberculous mycobacteria (NTM) than the TST. This is because the EC skin test only measures the immune response to two proteins that are found in the *Mycobacterium* tuberculosis bacteria, while the TST measures the immune response to a variety of proteins, including some that are also found in BCG and NTM (5, 15, 16). An increase in false-positive results from TSTs could lead to more students receiving preventive treatment, which might aggravate social panic and exacerbate the difficulty of controlling the epidemic.

The Chinese government recommends that individuals under the age of 15 be screened for LTBI and that those who are found to have LTBI have a CXR (6). We support this strategy. However, in some special cases, such as when the index case is bacteriologically positive for tuberculosis and the rate of LTBI is higher than the average for the local same-age group, CXR should be considered for these individuals. CXR is preferred to CT due to its lower radiation exposure. However, the diagnosis of tuberculosis in children is often challenging due to its subclinical symptoms, difficulty in providing specimens, and low bacterial loads (17–19). Only 20%−50% of children with tuberculosis are diagnosed with bacteriologically positive tuberculosis. This means that most children with tuberculosis are diagnosed based on imaging findings (18, 20). CT scans are more sensitive than CXRs for detecting the radiological signs of tuberculosis. This is because CT scans have a higher resolution and can better distinguish between different types of tissue. Additionally, CT scans are less susceptible to inter-observer variability, meaning that different radiologists are more likely to agree on the findings of a CT scan than on the findings of a CXR (19, 21, 22). Furthermore, tuberculous lymphadenopathy is common in children with tuberculosis, but CXRs are not very good at detecting mediastinal and hilar lymphadenopathy (23–25). In comparison, CT is considered the gold standard for showing enlarged lymph nodes in children with primary tuberculosis. CT can identify the early features of tuberculosis (lymphadenopathy, nodules, small pleural effusion) much earlier than CXR (18, 22, 23). In this outbreak, CXRs only identified 5 tuberculosis patients, while CT scans identified all 9. If CT scans had not been used, 4 patients would have been missed, who could have become new sources of infection and led to further transmission. Therefore, we cannot ignore the role of CT scans in the diagnosis of early tuberculosis, even though they expose patients to more radiation.

Retesting students with negative LTBI results is not mandatory when there are only one or two students with tuberculosis (6). In some special cases, such as when the index case is bacteriologically positive for tuberculosis and the rate of LTBI is higher than the average for the local same-age group, secondary screening may be necessary. Tuberculosis is a chronic infectious disease with a window period of up to 8 weeks (the time between infection and when the immunological response becomes measurable), which means that additional time is required to detect infection status. Both TST and the IGRAs have window periods (26, 27), therefore, there is a possibility of misclassification of infection status, as some children with LTBI may not be detectable. The index case being bacteriologically positive means that they are more infectious, and a higher LTBI rate means that there may be a delay in diagnosis. Delayed diagnosis is a major factor leading to TB outbreaks in schools, as prolonged exposure to the bacteria can lead to more severe disease (28, 29). The rate of LTBI reached 26%, which was much higher than the local average of 10%. Secondary screening is recommended 2–3 months after the first screening to identify those who may have become infected since the initial screening.

We observed another intriguing phenomenon that LTBI rate determined by the EC skin test was as high as 70.5% in the same class with the index cases. Typically, LTBI rates using EC ranged from 3% to 15%, and with the TST method, they varied from 5% to 25% in students based on previous studies (30–33). Head-to-head comparisons involving TST, IGRA, and ECs demonstrated excellent concordance between EC and IGRA, although the concordance between EC and TST showed a slight decrease (5, 34). The underlying reasons for this phenomenon are multifaceted. One possibility is that the EC skin test and TST were conducted at different times without a direct head-to-head comparison. EC, TST, and IGRA all have a window period (the time between individuals being infected and when the immunological response becomes measurable) of up to 8 weeks, suggesting that additional time may be necessary to accurately assess the infection status of a case (1). Another plausible explanation is that prolonged contact with the index case and secondary cases might result in new cases of LTBI. Additionally, among all contacts, the LTBI rate determined by the EC skin test was 18.5%, suggesting that the elevated rate may be attributed to close contact with index cases. Moreover, the unique educational environment, characterized by intense pressure from the examination system, could contribute to increased infection risks among students (11). The overall rate of LTBI was comparable to a school tuberculosis outbreak in Norway (15.4% by IGRA) (35) and a previous study of mine (23.4% by TST) (11). Furthermore, none of the individuals with positive EC results developed active tuberculosis after receiving TPT during the 1-year follow-up. This outcome suggests that treating LTBI in populations at an elevated risk of tuberculosis is an effective measure for tuberculosis control (36).

A key constraint of our study was the inability to conduct whole-genome sequencing (WGS) analysis, hindering the identification of transmission networks between index and secondary cases. Unfortunately, we lacked positive isolates of *Mycobacterium* tuberculosis for this analysis. Another limitation arose from the non-simultaneous administration of EC skin test and TST. While EC successfully detected all 9 suspected active tuberculosis cases identified through sputum and/or CT scans, TST identified only 3. Although it may suggest that EC outperformed TST, the potential bias introduced by the differing timing of the tests should be acknowledged.

## Conclusions

In summary, the novel EC skin test performed well in the school tuberculosis outbreak. However, in some special cases, such as when the index case is bacteriologically positive for tuberculosis and the rate of LTBI is higher than the average for the local same-age group, secondary screening is recommended 2–3 months after the first screening. Additionally, we cannot ignore the role of CT in the diagnosis of early student tuberculosis.

## Data availability statement

The datasets presented in this article are not readily available because the databases in this study were from a school tuberculosis outbreak. Requests to access the datasets should be directed to LZ, lilyam0921@163.com.

## Ethics statement

The requirement of ethical approval was waived by the Ethics Committee of Center for Disease Control and Prevention of Jiangsu Province for the studies involving humans because all data were from epidemic investigation, so ethics approval was not needed in this study. The studies were conducted in accordance with the local legislation and institutional requirements. Written informed consent for participation in this study was provided by the participants' legal guardians/next of kin.

## Author contributions

LZ: Funding acquisition, Project administration, Writing—review & editing. PL: Formal analysis, Writing—original draft, Writing—review & editing. JX: Formal analysis, Investigation, Writing—original draft. RW: Investigation, Writing—original draft. XG: Investigation, Writing—original draft. QL: Formal analysis, Investigation, Writing—original draft. XD: Investigation, Writing—original draft. WL: Investigation, Writing—original draft.
